# Gemcitabine plus best supportive care (BSC) vs BSC in inoperable non-small cell lung cancer – a randomized trial with quality of life as the primary outcome

**DOI:** 10.1054/bjoc.2000.1307

**Published:** 2000-07-24

**Authors:** H Anderson, P Hopwood, R J Stephens, N Thatcher, B Cottier, M Nicholson, R Milroy, T S Maughan, S J Falk, M G Bond, P A Burt, C K Connolly, M B McIllmurray, J Carmichael

**Affiliations:** Wythenshawe Hospital, Manchester; CRC Psychological Medicine Group, Christie Hospital NHS Trust, Manchester; Cancer Division, MRC Clinical Trials Unit, London; Department of Medical Oncology, Christie CRC Research Centre, Christie Hospital NHS Trust, Manchester; Clatterbridge Hospital, Bebbington, Liverpool; Aberdeen Royal Infirmary, Aberdeen; Stobhill NHS Trust, Glasgow; Velindre Hospital, Whitchurch, Cardiff; Bristol Oncology Centre, Bristol; Cookridge Hospital, Leeds; Christie Hospital NHS Trust, Manchester; Darlington Memorial Hospital, Darlington; Lancaster Royal Infirmary, Lancaster; City Hospital, Nottingham, UK

**Keywords:** gemcitabine, BSC, NSCLC, quality of life

## Abstract

Three hundred patients with symptomatic, locally advanced or metastatic NSCLC not requiring immediate radiotherapy were enrolled into this randomized multicentre trial comparing gemcitabine + BSC vs BSC alone. Patients allocated gemcitabine received 1000 mg/m^2^on days 1, 8 and 15 of a 28-day cycle, for a maximum of six cycles. The main aim of this trial was to compare patient assessment of a predefined subset of commonly reported symptoms (SS14) from the EORTC QLQ-C30 and LC13 scales. The primary end-points were defined as (1) the percentage change in mean SS14 score between baseline and 2 months and (2) the proportion of patients with a marked (≥ 25%) improvement in SS14 score between baseline and 2 months sustained for ≥4 weeks. The secondary objectives were to compare treatments with respect to overall survival, and multidimensional QL parameters.The treatment groups were balanced with regard to age, gender, Karnofsky performance status (KPS) and disease stage (40% had metastatic disease). The percentage change in mean SS14 score from baseline to 2 months was a 10% decrease (i.e. improvement) for gemcitabine plus BSC and a 1% increase (i.e. deterioration) for BSC alone (*P* = 0.113, two-sample *t* -test). A sustained (≥ 4 weeks) improvement (≥25%) on SS14 was recorded in a significantly higher proportion of gemcitabine + BSC patients (22%) than in BSC alone patients (9%) (*P* = 0.0014, Pearson’s chi-squared test). The QLQ-C30 and L13 subscales showed greater improvement in the gemcitabine plus BSC arm (in 11 domains) than in the BSC arm (one symptom item). There was greater deterioration in the BSC alone arm (six domains/items) than in the gemcitabine + BSC arm (three QL domains). Tumour response occurred in 19% (95% CI 13–27) of gemcitabine patients. There was no difference in overall survival: median 5.7 months (95% CI 4.6–7.6) for gemcitabine + BSC patients and 5.9 months (95% CI 5.0–7.9) (log-rank, *P* = 0.84) for BSC patients, and 1-year survival was 25% for gemcitabine + BSC and 22% for BSC. Overall, 74 (49%) gemcitabine + BSC patients and 119 (79%) BSC patients received palliative radiotherapy. The median time to radiotherapy was 29 weeks for gemcitabine + BSC patients and 3.8 weeks for BSC. Patients treated with gemcitabine + BSC reported better QL and reduced disease-related symptoms compared with those receiving BSC alone. These improvements in patient-assessed QL were significant in magnitude and were sustained. © 2000 Cancer Research Campaign


*British Journal of Cancer* (2000) **83**(4), 447–453
© 2000 Cancer Research Campaign
doi: 10.1054/ bjoc.2000.1307, available online at http://www.idealibrary.com on IDEAL®

### Gemcitabine plus best supportive care (BSC) vs BSC in inoperable non-small cell lung cancer – a randomized trial with quality of life as the primary outcome

**H Anderson[1], P Hopwood[2], RJ Stephens[3], N Thatcher[4], B Cottier[5], M Nicholson[6], R Milroy[7], TS Maughan[8], SJ Falk[9], MG Bond[10], PA Burt[11], CK Connolly[12], MB McIllmurray[13] and J Carmichael[14] on behalf of the UK NSCLC Gemcitabine Group**

[1]Wythenshawe Hospital, Manchester; [2]CRC Psychological Medicine Group, Christie Hospital NHS Trust, Manchester; [3]Cancer Division, MRC Clinical Trials Unit, London; [4]Department of Medical Oncology, Christie CRC Research Centre, Christie Hospital NHS Trust, Manchester; [5]Clatterbridge Hospital, Bebbington, Liverpool; [6]Aberdeen Royal Infirmary, Aberdeen; [7]Stobhill NHS Trust, Glasgow; [8]Velindre Hospital, Whitchurch, Cardiff; [9]Bristol Oncology Centre, Bristol; [10]Cookridge Hospital, Leeds; [11]Christie Hospital NHS Trust, Manchester; [12]Darlington Memorial Hospital, Darlington; [13]Lancaster Royal Infirmary, Lancaster; [14]City Hospital, Nottingham, UK

**Summary** Three hundred patients with symptomatic, locally advanced or metastatic NSCLC not requiring immediate radiotherapy were enrolled into this randomized multicentre trial comparing gemcitabine + BSC vs BSC alone. Patients allocated gemcitabine received 1000 mg/m$^2$ on days 1, 8 and 15 of a 28-day cycle, for a maximum of six cycles. The main aim of this trial was to compare patient assessment of a predefined subset of commonly reported symptoms (SS14) from the EORTC QLQ-C30 and LC13 scales. The primary end-points were defined as (1) the percentage change in mean SS14 score between baseline and 2 months and (2) the proportion of patients with a marked (≥ 25%) improvement in SS14 score between baseline and 2 months sustained for ≥4 weeks. The secondary objectives were to compare treatments with respect to overall survival, and multidimensional QL parameters.The treatment groups were balanced with regard to age, gender, Karnofsky performance status (KPS) and disease stage (40% had metastatic disease). The percentage change in mean SS14 score from baseline to 2 months was a 10% decrease (i.e. improvement) for gemcitabine plus BSC and a 1% increase (i.e. deterioration) for BSC alone ($P$ = 0.113, two-sample $t$-test). A sustained (≥ 4 weeks) improvement (≥25%) on SS14 was recorded in a significantly higher proportion of gemcitabine + BSC patients (22%) than in BSC alone patients (9%) ($P$ = 0.0014, Pearson's chi-squared test). The QLQ-C30 and L13 subscales showed greater improvement in the gemcitabine plus BSC arm (in 11 domains) than in the BSC arm (one symptom item). There was greater deterioration in the BSC alone arm (six domains/items) than in the gemcitabine + BSC arm (three QL domains). Tumour response occurred in 19% (95% CI 13–27) of gemcitabine patients. There was no difference in overall survival: median 5.7 months (95% CI 4.6–7.6) for gemcitabine + BSC patients and 5.9 months (95% CI 5.0–7.9) (log-rank, $P$ = 0.84) for BSC patients, and 1-year survival was 25% for gemcitabine + BSC and 22% for BSC. Overall, 74 (49%) gemcitabine + BSC patients and 119 (79%) BSC patients received palliative radiotherapy. The median time to radiotherapy was 29 weeks for gemcitabine + BSC patients and 3.8 weeks for BSC. Patients treated with gemcitabine + BSC reported better QL and reduced disease-related symptoms compared with those receiving BSC alone. These improvements in patient-assessed QL were significant in magnitude and were sustained. © 2000 Cancer Research Campaign

**Keywords**: gemcitabine; BSC; NSCLC; quality of life

The role of chemotherapy in non-small cell lung cancer (NSCLC) remains controversial. Although a meta-analysis of randomized trials in the treatment of inoperable NSCLC has shown a small survival advantage for cisplatin regimens over best supportive care (BSC) (NSCLCCG, 1995), treatment remains largely palliative in intent. In these circumstances, there is a need to balance the benefits of palliative treatment against toxicity, (Brinkley, 1985). Despite the widely published endorsement of the need to assess patients' quality of life (QL) and the availability of appropriate QL measures, there has been little real commitment to the inclusion of QL assessment in clinical trials of lung cancer (Hopwood, 1997).

Improvements occur in disease-related symptoms with chemotherapy, such as MVP mitomycin C, vinblastine, cisplatin (Fernandez et al, 1989; Hardy et al, 1989) and MIC mitomycin C, ifosfamide, cisplatin (Cullen 1993), and the relative effectiveness of these palliative regimens has been compared (Thatcher et al, 1995). It was reported that more patients gain symptomatic benefit than is suggested by objective tumour response rate. However, a systematic assessment of QL was not included in these studies (Thatcher et al, 1997).

Gemcitabine has been evaluated in several phase II studies in NSCLC. These have shown independently validated objective response rates ranging from 18–26%, with median survival times of 6.2–12.3 months (Noble and Goa, 1997). Furthermore, data suggest that gemcitabine is both better tolerated than cisplatin plus

*Received 29 November 1999*
*Revised 23 March 1999*
*Accepted 13 April 2000*

*Correspondence to:* H Anderson etoposide (Noble and Goa, 1997) and improves disease-related symptoms and performance status (Thatcher et al, 1997). Gemcitabine was therefore an appropriate agent for comparison against a no-drug control arm using QL outcomes.

In defining a suitable QL end-point, reference was made to the analysis of QL data from a randomized trial of treatment in NSCLC conducted by the Medical Research Council Lung Cancer Working Party, which indicated that respiratory symptoms were necessary but insufficient for comparing treatment regimens (Hopwood et al, 1995). In 423 patients assessed, the average number of symptoms at presentation was 13 (two severe, three moderate and eight mild) and the 10 most prevalent symptoms included not only respiratory symptoms, but those reflecting general debility (e.g. tiredness, lack of appetite) and psychological distress.

If improvement in the well-being of lung cancer patients is to be adequately assessed, the primary trial end-point must include the impact of treatment on the wide range of symptoms experienced. Comparison of symptom recording by patients and their treating doctors has confirmed that doctors underestimate even the most common symptoms in patients with lung cancer (such as shortness of breath) (Stephens et al, 1997). QL assessments should be based on patients' own ratings. Careful thought should be given to the optimal time-point for measuring QL outcomes, and to the criteria on which QL should be compared. This is particularly difficult given the multidimensional nature of QL and the need to compare parameters that may change in different directions at different points in time. Attrition due to patient death and low compliance in poor performance status patients will limit the amount of evaluable QL data and missing data will create difficulties in analysis (Hopwood, 1996). Moreover, there is no agreed definition of palliation for application in this context (Stephens et al, 1999). These problems were addressed in the design and implementation of this trial of chemotherapy plus BSC vs BSC alone. To our knowledge, this was the first lung cancer treatment trial designed to rely on QL end-points as the primary outcome measure in a general population of NSCLC patients. This trial opened in December 1994. Since then a randomized trial of the Italian Study Group opened in April 1996.

### METHODS

### Patients

Patients with histologically or cytologically proven NSCLC were eligible if they were previously untreated and had symptomatic locally advanced or metastatic disease which was not amenable to curative surgery or radiotherapy. Patients had to have a Karnofsky performance status of 60–90, clinically measurable disease (uni- or bidimensionally measurable) and an estimated life expectancy of at least 4 weeks. Patients were excluded from entry into the study if they needed urgent radiotherapy, had brain metastases, inadequate bone-marrow reserve (leucocyte count $<3.5 \times 10^9$ $l^{-1}$, platelets $<100 \times 10^9$ $l^{-1}$, and haemoglobin $<100$ g $l^{-1}$), or inadequate liver function (bilirubin >3 times above normal range; alanine transaminase or aspartate transaminase >3 times normal (or >5 times normal in patients with known liver metastases)). Patients had to be willing and able to complete QL questionnaires and give written, informed consent. Local ethics committees' approval had to be obtained.

### Treatment

Patients allocated to gemcitabine + BSC were treated as outpatients with 1000 mg $m^{-2}$ intravenous gemcitabine over 30 min on days 1, 8 and 15 of each 28-day cycle for up to six cycles of treatment. Patients were seen weekly during chemotherapy and a full blood count was performed weekly during the first cycle and every 2 weeks thereafter. In the event of specified World Health Organization (WHO) grade 3 or 4 toxicities, dose reductions or omissions were made according to a standardized protocol. Chemotherapy was stopped in the event of tumour progression, toxicity or patient request to discontinue therapy. Patients allocated to BSC were seen in the clinic every 4 weeks and were treated symptomatically; any palliative treatment could be used as clinically indicated, ideally excluding chemotherapy.

### Randomization and masking

Computer generated randomization was performed centrally by telephone and patients were stratified for the 25 treatment centres, performance status (KPS 80–90 and 60–70), and disease extent (locoregional vs metastatic), using an algorithm described by Pocock and Simon (1975).

### Objectives and end-points

The primary objective was to compare gemcitabine plus BSC to BSC alone with respect to patient assessment of a predefined subset of 14 commonly reported symptoms (SS14) (see Table 1) from standardized QL measures, the EORTC QLQ-C30 (Aaronson et al, 1993) and LC13 (Bergman et al, 1994). The end-points used to assess change in symptoms were: the percentage change in mean SS14 score from randomization to 2 months; and the proportion of patients with sustained improvement of SS14 score at 2 months, defined as a ≥ 25% reduction from baseline sustained from month 1 to month 2, and/or from month 2 to month 3.

The secondary objectives were to compare treatment groups with respect to (1) overall survival, and (2) all QL parameters. In addition, (3) the objective tumour response rate amongst patients receiving gemcitabine plus BSC was assessed. The corresponding end-points were:

1. Time to death. Patients were followed up until the time of death. Patients alive at the time of data analysis were censored at the last date they were known to be alive.
2. Patient-assessed QL using all the subscales and symptom items on the QL measures. Changes from baseline to 2, 4 and 6 months were calculated in terms of the proportion of patients who improved or deteriorated. Since small differences were unlikely to reflect clinical benefit, interest was focused on those subscales or symptom items that showed a ≥ 10% between-treatment difference in the number of patients who improved or deteriorated.
3. Objective tumour response rate (for the gemcitabine plus BSC arm only). Tumour response was defined according to WHO (1979) criteria.

### Assessments

Three additional symptom items were included to assess possible gemcitabine side-effects (skin rash/itchiness, ankle swelling,

© 2000 Cancer Research Campaign flu-like symptoms). These symptom items were formatted in the same way as other items on the EORTC subscales. Patients completed the EORTC QLQ-C30 and LC13 questionnaires every 4 weeks, prior to their clinical assessment.

A predetermined subset of items (SS14) from the above QL scales was used for the analysis of the primary end-point (Table 1). The SS14 included disease-specific items plus the other most frequently reported symptoms in patients with NSCLC identified in another patient cohort (Hopwood et al, 1995).

### Statistical methods

The study was designed to recruit 300 patients with 150 patients in each arm. The percentage change in the mean score of the SS14 items in each randomized group, from baseline to 2 months, was compared using a two sample *t*-test. The trial was designed so that the sample size of 150 patients per arm would provide 90% power to detect a difference of 0.4 SD at the 5% significance level. The difference in sustained symptom improvement rates was assessed using Pearson's chi-squared test. Overall survival curves were produced using the Kaplan–Meier method, and were compared using the log-rank test. Baseline QL forms were only included if completed on or before randomization, but acceptable time windows of ±1 week were permitted around QL assessment points of 2, 4 and 6 months.

### RESULTS

### Trial profile

Three hundred patients from 25 centres were enrolled over 16 months between December 1994 and May 1996. All 300 patients enrolled were eligible for randomization, with 150 patients in each arm (Figure 1). One patient was subsequently found to have mesothelioma but the results presented are on an intent-to-treat basis. Patients were well matched for pre-treatment characteristics (Table 2): age, gender, Karnofsky performance status (KPS) and stage (40% had metastatic disease).

**Table 1** SS14 symptom scale

| Original number in QLQ-C30 and LC13 scales | New subset number | Question |
|---|---|---|
| 31 | 1 | How much did you cough? |
| 32 | 2 | Did you cough blood? |
| 33 | 3 | Were you short of breath when you rested? |
| 34 | 4 | Were you short of breath when you walked? |
| 35 | 5 | Were you short of breath when you climbed stairs? |
| 40 | 6 | Have you had pain in your chest? |
| 41 | 7 | Have you had pain in your arm or shoulder? |
| 42 | 8 | Have you had pains in other parts of your body? |
| 12 | 9 | Have you felt weak? |
| 18 | 10 | Were you tired? |
| 11 | 11 | Have you had trouble sleeping? |
| 22 | 12 | Did you worry? |
| 13 | 13 | Have you lacked appetite? |
| 16 | 14 | Have you been constipated? |

### Compliance

Sixty-seven per cent of patients randomized were evaluable for analysis of QL data with respect to the primary end-point. The reasons for patients being unevaluable for QL are summarized in Figure 1. Using available QL data, baseline scores for all QL subscales and items were compared for the 201 evaluable patients and 96 patients who did not qualify for this primary analysis. Unevaluable patients had a greater symptom burden and poorer function as indicated by a ≥ 10-point difference in mean and/or median scores for the fatigue and social functioning subscales, appetite loss, constipation and pain (other than chest or shoulder pain), but were comparable on all other QL domains. Evaluable patients had greater symptom burden in the cognitive domain.

### Therapy received

Patients allocated to gemcitabine received a median of three cycles (mean 3.2, range 0–6) and 29 (19%) of 150 gemcitabine patients received six cycles as planned. The mean dose of gemcitabine delivered in this study was 887 mg $m^{-2}$, which represents 89% of the planned dose of 1000 mg $m^{-2}$. Eight per cent of injections were omitted and 3% reduced. At disease progression, few patients received chemotherapy: five BSC patients who progressed at 2, 8.5, 29, 33 and 52 weeks (four had cisplatin combinations; one had gemcitabine); and three gemcitabine plus BSC patients, who progressed at 23, 45 and 94 weeks (all re-treated with gemcitabine). The patient who received other chemotherapy at 2 weeks was not eligible for inclusion in the quality-of-life analysis.

Although patients with a need for urgent radiotherapy were excluded from entry into the study, 74 (49%) patients on gemcitabine plus BSC vs 119 (79%) BSC patients received palliative radiotherapy. At 2 months, 13 (9%) of gemcitabine plus BSC patients vs 87 (58%) of BSC patients had received radiotherapy. The median time to radiotherapy was significantly longer for gemcitabine plus BSC (29.1 weeks) than for BSC (3.8 weeks) ($P < 0.001$). The indications for palliative radiotherapy were the same in each arm of the study. Of patients receiving radiotherapy in the gemcitabine plus BSC arm 54% had the mediastinum and 22% had the chest treated. In the BSC arm radiotherapy was given to the mediastinum in 56% and chest in 34% cases.

### Primary end-point: SS14

All items on the SS14 were scored in the same direction, with higher scores representing higher symptom burden. The primary

**Table 2** Characteristics of randomized patients

| | Gemcitabine plus BSC | BSC |
|---|---|---|
| Patients randomized | 150 | 150 |
| Female:male | 51:99 | 59:91 |
| Median age (range) | 65 (37–82) | 64 (32–83) |
| Karnofsky performance status | | |
| 60 | 57 | 52 |
| 70 | 48 | 58 |
| 80 | 35 | 32 |
| 90 | 10 | 8 |
| Stage | | |
| Locally advanced | 88 (58.7%) | 92 (61.3%) |
| Metastatic | 62 (41.3%) | 58 (38.7%) |

© 2000 Cancer Research Campaign

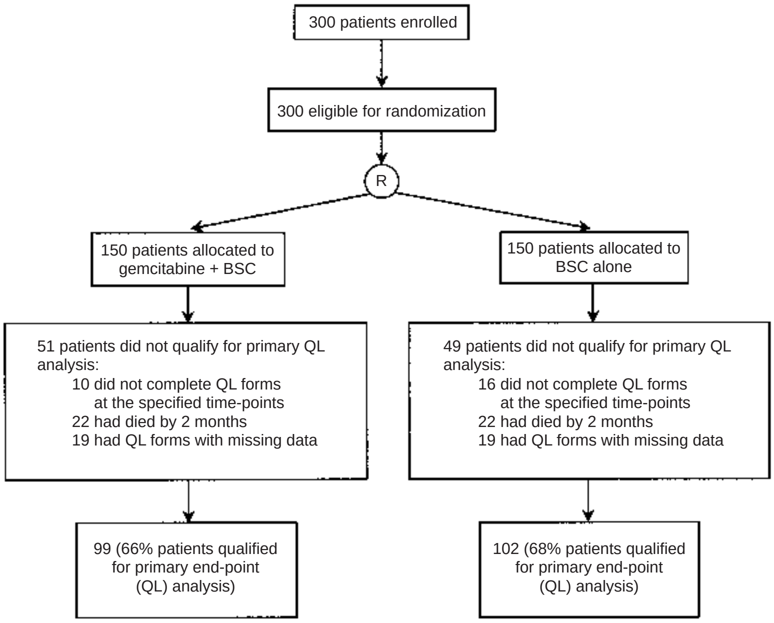

**Figure 1** Trial profile

**Table 3** Patient-assessed symptom scale (SS14)

| | **Gemcitabine plus BSC** | **BSC** | ***P*-values** |
|---|---|---|---|
| Percentage change in SS14 scores from baseline–2 months | | | |
| Patients evaluable | 99 | 102 | |
| Mean % change | –10.2 | +1.1 | 0.113[a] |
| Analysis of sustained (≥4 weeks) SS14 improvement (≥25%) | | | |
| Patients with sustained improvement | 33 (22%) | 13 (9%) | 0.0014[b] |
| Patients with no sustained improvement | 117 (78%) | 137 (91%) | |
| SS14 improvement (≥25%) at 2, 4 and 6 months | | | |
| 2 months | | | |
| Patients evaluable | 99 | 102 | |
| Patients improved | 38 (38%) | 25 (24%) | 0.065[b] |
| 4 months | | | |
| Patients evaluable | 68 | 61 | |
| Patients improved | 30 (44%) | 15 (25%) | 0.015[b] |
| 6 months | | | |
| Patients evaluable | 36 | 40 | |
| Patients improved | 11 (31%) | 9 (22%) | 0.644[b] |

[a]Two-sample *t*-test; [b]Pearson's chi-squared test objective end-point data were as follows: (1) the percentage change in mean SS14 score from baseline to 2 months was –10% (i.e. improvement) for gemcitabine + BSC and +1% (i.e. deterioration) for BSC ($P$ = 0.113) (Table 3); (2) Sustained (≥ 4 weeks) improvement (≥ 25%) in SS14 score was recorded in a significantly higher proportion of gemcitabine plus BSC patients (22%) than in BSC patients (9%) ($P$ = 0.0014, Pearson's chi-squared test) (Table 3).

Improvement (≥ 25%) in SS14 score at 2, 4 and 6 months occurred in a greater proportion of patients treated with gemcitabine plus BSC than in those receiving BSC alone (Table 3). Numbers of patients at 4 and 6 months were insufficient to estimate sustained improvement.

### Quality of life

All evaluable EORTC QLQ-C30 and LC13 data were compared between regimens at 2 and 4 months. Since small differences were unlikely to reflect clinical benefit, interest was focused on those subscales or symptom items that showed a ≥ 10% between-treatment difference in the number of patients who improved or deteriorated.

At 2 months (Figure 2A), of the 25 variables analysed, six showed between-treatment differences in improvements that were ≥ 10%: five of the improvements were greater for gemcitabine plus BSC (emotional functioning, pain-symptom scale, chest pain, cough, fatigue), whereas one was greater for BSC (dyspnoea). At

*© 2000 Cancer Research Campaign*

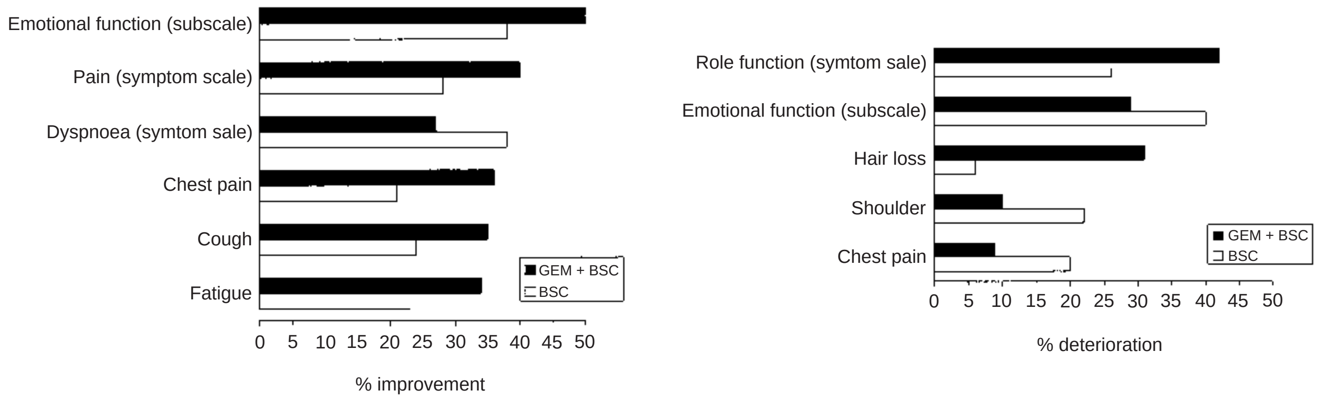

**Figure 2** EORTC QLC-C30 and LC13 subscales and items that showed ≥10% between-treatment differences in the proportion of patients (**A**) reporting improvement from baseline to 2 months (B) reporting deterioration from baseline to 2 months

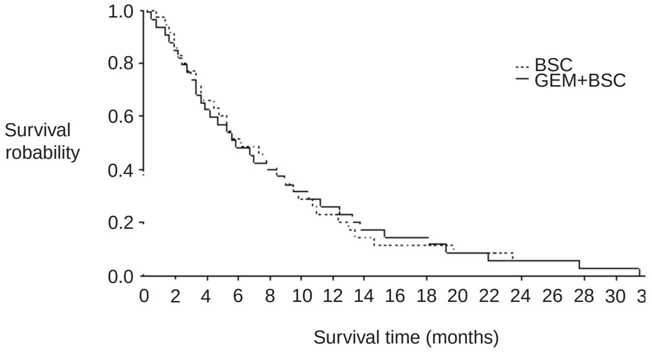

**Figure 3** Kaplan–Meier survival curves for patients in the gemcitabine plus BSC and BSC arms

2 months (Figure 2B), five variables showed between-treatment differences in deterioration that were ≥ 10%: two of the deteriorations were greater for gemcitabine plus BSC (role function and hair loss), whereas three were greater for BSC (chest pain, shoulder pain, emotional functioning).

Similarly, at 4 months, six variables showed between-treatment differences in improvements that were ≥ 10%. All six improvements were greater for gemcitabine plus BSC (chest pain, shoulder pain, emotional functioning, role domain, social domain, financial impact). Also at 4 months, four variables showed between-treatment differences in deterioration that were ≥ 10%: one of the deteriorations was greater for gemcitabine plus BSC (hair loss), whereas three deteriorations were greater for BSC (social domain, pain-symptom scale, constipation).

Improvements in KPS (lasting at least 4 weeks) were seen in 20.3% of gemcitabine plus BSC patients and in 12.3% of BSC patients ($P$ = 0.073).

### Tumour response

Fifteen gemcitabine plus BSC patients did not have tumour measurements available due to insufficient therapy (11 patients), lack of uni- or bidimensional lesions (three patients), and a diagnosis of mesothelioma (one patient). Of 135 patients with at least two assessments of tumour size, 25 patients had objective responses (overall response rate, 18.5%; 95% CI 13–26).

### Survival

As of 4 June 1998, 13 patients were still alive and median follow-up for these survivors was 25.3 months (range 1.3–40.3 months). There was no difference in survival between the two arms (Figure 3). Median survival was 5.7 months for gemcitabine plus BSC patients (95% CI 4.6–7.6) and 5.9 months for BSC (95% CI 5.0–7.9) (log-rank, $P$ = 0.84). Estimated 1-year survival rate was 25% for gemcitabine plus BSC and 22% for BSC. Two-year survival rate was 6% for gemcitabine plus BSC and 7% for BSC.

### Toxicity

The incidence of WHO grade 3 and 4 toxicity in gemcitabine plus BSC patients was low, as has been reported in phase II studies of single-agent gemcitabine (Aapro et al 1998): neutropenia 13%, infection 0.7%, thrombocytopenia 2%, nausea and vomiting 9%, lethargy 6%, rash 4% and pulmonary toxicity 3%. Patient-reported symptoms used to assess chemotherapy toxicity showed, as expected, that patients on the gemcitabine plus BSC arm at 2 months had increased prevalence of hair loss (31% vs 6%), ankle swelling (30% vs 11%) and flu-like symptoms (32% vs 15%), but not skin rash (13% vs 16%).

Gemcitabine is a radiosensitizer when given concurrently with radiation. Radiation was not given concurrently with gemcitabine in this trial. The Radiation Therapy Oncology Group (RTOG) toxicity (Cox et al, 1995) was low, grade 3 and 4 pharyngeal/oesophageal and skin toxicity was ≤ 2% in each arm. RTOG grade 3 and 4 pulmonary toxicity occurred in 4% of BSC patients who received radiotherapy, but in none of the patients in the gemcitabine plus BSC arm who subsequently received radiotherapy.

### DISCUSSION

The similar survival in the two treatment arms of this randomized trial highlight the importance of balancing the QL costs and benefits of chemotherapy in the palliative treatment of NSCLC. Yet, while the need to evaluate palliative treatments in this way has been widely advocated, there has been a disappointing level of commitment to the necessary assessments of QL in cancer clinical trials (Batel-Copel et al, 1997). Our trial, commenced in 1994, attempts to address this important issue, using QL parameters as the primary outcome, in order to give a clear focus to the benefit and impact of treatment in these patients. In 1996 a similar approach was used in a randomized trial of vinorelbine vs BSC in elderly patients with advanced NSCLC (ELCVISG, 1999), QL (assessed using the same scales) and survival were primary outcomes. Between-treatment differences in the QL domains were reported using a complex analysis method to adjust for the problem of attrition. However, it is difficult to tease out the level of clinical benefit from these data as there was significantly more toxicity with hair loss, constipation and peripheral neuropathy on QL assessment. It is hoped that other trial groups will add to the experience of QL assessment in the palliative setting.

Forty per cent of patients in our trial had stage IV disease, and 1-year survival was 25% for gemcitabine plus BSC and 22% for BSC alone. In a meta-analysis, 1-year survival was 16% for BSC and 26% for patients treated with cisplatin therapy (NSCLCCG, 1995; Stewart et al, 1994). Our result was in keeping with other studies. The response rate of 19% was comparable with the lower range of results from phase II studies of gemcitabine, and probably reflects efficacy in a less-selected patient group. Indeed, for phase II studies, entry criteria usually stipulate estimated life expectancy of ≥ 12 weeks, whereas in this study life expectancy had to be ≥ 4 weeks. We were surprised at the number of patients in the control arm requiring early radiotherapy, given that an urgent need for radiotherapy made patients ineligible for randomization. At the 2-month QL assessment 58% BSC patients had received radiotherapy compared with 9% gemcitabine-treated patients.

The results of this study confirm a significant and sustained improvement in the most prevalent symptoms in NSCLC patients treated with gemcitabine plus BSC, although the level of improvement varied considerably between different symptom areas, supporting the need for a broad approach to treatment evaluation. Disappointingly, breathlessness was not well palliated by gemcitabine. This may have been due to increased activity with improvement in performance status and reduced lethargy, rather than any pulmonary toxicity, for which the incidence was low (3% gemcitabine plus BSC patients experienced WHO grade 3 and 4 toxicity and 4% BSC patients treated with radiotherapy had RTOG grade 3 and 4 toxicity). It is of concern that overall, only one-fifth to one-third of these trial patients gained relief from common disease-related symptoms such as chest pain, cough or dyspnoea. Interestingly, fatigue improved in both arms of the trial, despite frequent expectations that chemotherapy and/or radiotherapy will affect this adversely. Gemcitabine plus BSC had the most marked benefit on emotional functioning, suggesting that active, systemic treatment is more acceptable to patients than is often assumed.

The practical problems for investigators researching QL in the palliative setting have been well described (Hopwood et al 1994; Hopwood, 1996; Thatcher et al, 1997). Of particular concern is patient attrition and the risk of bias if ill patients are unable to complete QL forms or staff are unwilling to approach them (Hopwood et al, 1998). Careful attention was given to these aspects during the planning and implementation of this study to optimize data collection, and considerable additional resources were required to achieve this. The proportion of patients with evaluable baseline and 2-month data (66%) is probably realistic for any study of this type (given minimal expected survival of 1 month), and an improvement on others (Bernhard and Gelber, 1998). Reassuringly, missing data do not appear to have introduced bias into the resulting comparison. Moreover, the application of tight time-windows enabled us to keep random and non-random bias to a minimum, and we feel confident that the results are an accurate reflection of the patients assessed.

Controversy continues as to whether QL outcomes should be summarized, to simplify analysis and reporting (Barsevik et al, 1997; Billingham et al, 1997) at the risk of being clinically uninterpretable, or remain disaggregated, to provide a breadth of information which may, however, be difficult to present and absorb (MRC LCWP 1996*a*; 1996*b*; Harper et al, 1997). Both approaches are numerically driven and may suffer from lack of clear indicators of clinical benefit. In this trial we created a short-scale of the most prevalent symptoms, for the purpose of analysis, to address the need for clinical relevance in demonstrating palliation in several symptom domains without reliance on multiple subscales. Comparing the proportions of patients improving by a predetermined amount on this scale enabled us to provide a clinically interpretable outcome. While not a perfect solution, we think this method warrants replication.

The collection of QL data for use as a primary outcome proved feasible within a UK multicentre setting, but the resources needed to ensure good-quality QL data are considerable. Funding agencies need to be prepared to support these costs in clinical trials' budgets, if reliable QL outcomes are required, and if the investment of the past two decades in QL methodology is to bear fruit. Although it may have been desirable to measure the primary endpoint later in this trial, the further expected attrition would have required a substantially increased sample size.

Patient-rated QL data showed that improvements were significant in duration and magnitude in the chemotherapy arm, together with improved performance status as measured by clinicians and a reduced need for palliative radiotherapy. Since our trial commenced we are aware of one other study which has used quality of life as a primary outcome measure (ELCVISG, 1999). We would advocate this approach in other palliative trials, in order to address the impact on important aspects of patient well-being and challenge inappropriate assumptions.

The results of this study showing quality of life benefit, the Italian study showing improved survival, cognitive function, dyspnoea and pain in elderly patients, and a study from Billingham et al (1997) showing improved survival and quality of life score with cisplatin combination chemotherapy vs BSC, suggest that appropriate patients should be offered palliative chemotherapy rather than entered into randomized trials containing a best supportive care arm.

### ACKNOWLEDGEMENTS

We thank the following institutions and clinicians who entered patients into this study: Aberdeen Royal Infirmary (Dr M Nicholson); Airedale General Hospital, Keighley (Dr SM Crawford); Bristol Oncology Centre (Dr S Falk); City Hospital, Nottingham (Dr J Carmichael); Clatterbridge Hospital, Bebington (Dr B Cottier); Cookridge Hospital Chest Clinic, Leeds (Dr M Bond); Darlington Memorial Hospital (Dr CK Connolly);

 © 2000 Cancer Research Campaign

Freedom Fields Hospital, Plymouth (Dr S Kelly); Glasgow Royal Infirmary (Dr DJ Dunlop); Hull Royal Infirmary (Dr M Greenstone); Ipswich Hospital NHS Trust (Dr JS Morgan); King's Cross Hospital, Dundee (Dr JH Winter); Manchester, Christie and Wythenshawe Hospitals (Dr H Anderson, Professor N Thatcher, Dr P Burt); Middlesex Hospital, London (Dr SG Spiro); Pontefract General Infirmary (Dr MD Peake); Royal Lancaster Infirmary (Dr MB McIllmurray); Royal United Hospital, Bath (Dr H Newman); Southend District Hospital, Westcliff-on-Sea (Dr CWL Trask); St Bartholomew's Hospital, London (Dr RM Rudd); St George's Hospital, London (Dr J Mansi); Stobhill NHS Trust, Glasgow (Dr R Milroy); Velindre Hospital, Whitchurch, Cardiff (Dr T Maughan, Dr Paterson); Walsgrave Hospital, Coventry (Dr DP Dhillon); Weston Park Hospital NHS Trust, Sheffield (Dr DJ Radstone) and; Ysbyty Gwynedd, Bangor (Dr NSA Stuart).

This study was supported by a grant from Eli Lilly and Company, Indianapolis, USA.

### REFERENCES

Aapro MS, Martin C and Hatty S (1998) Gemcitabine: a safety review. *Anti-Cancer Drugs* **9**: 191–201

Aaronson NK, Ahmedzai S, Bergman B, Bullinger M, Cull A, Duez NJ, Filiberti A, Flechtner H, Fleisham SB and deHaes JC (1993) The EORTC QLQ-C30: a quality of life instrument for use in international clinical trials in oncology. *J Natl Cancer Inst* **85**: 365–376

Barsevik A, Langer C, Brunet D, Grindel C, Leighton J and Luckscheider C (1997) Correlation of quality of life (QOL) with survival, treatment response, and anemia in patients with advanced non-small cell lung cancer (NSCLC) treated with carboplatin and paclitaxel [abstract]. *Proc Am Soc Clin Oncol* **16**: 44a

Batel-Copel M, Kornblith AB and Batel PC (1997) Do oncologists have an increasing interest in the quality of life of their patients? A literature review of the last 15 years. *Eur J Cancer* **33**: 29–32

Bergman B, Aaronson NK, Ahmedzai S, Kaasa S and Sullivan M (1994) The EORTC QLQ-LC13: a modular supplement to the EORTC core quality of life questionnaire (QLQ-C30) for use in lung cancer clinical trials. *Eur J Cancer* **30A**(5): 635–642

Bernhard J and Gelber RD (eds) (1998) Workshop on missing data in quality of life research in cancer clinical trials: practical and methodological issues. *Stat Med* **17**: 5–7

Billingham LJ, Cullen MH, Woods AD and Chetiyawardana RC (1997) Mitomycin, ifosfamide and cisplatin (MIC) in non-small cell lung cancer: 3. Results of a randomised trial evaluating palliation and quality of life [abstract]. *Lung Cancer* **18**(Suppl 1): 9

Brinkley D (1985) Quality of life in cancer trials. *BMJ* **291**: 685–686

Cox JD, Stetz J and Pajak TF (1995) Toxicity criteria of the Radiation Therapy Oncology Group (RTOG) and the European Organization for Research and Treatment of Cancer (EORTC). *Int J Radiat Oncol Biol Phys* **31**(5): 1341–1346

Cullen MH (1993) The MIC regimen in non-small cell lung cancer. *Lung Cancer* **9**(Suppl 2): 81–89

Elderly Lung Cancer Vinorelbine Italian Study Group (1999) Effects of vinorelbine on quality of life and survival of elderly patients with advanced non-small cell lung cancer. *J Natl Cancer Inst* **91**: 66–72

Fernandez C, Rosell R and Abad-Esteve A et al (1989) Quality of life during chemotherapy in non-small cell lung cancer patients. *Acta Oncol* **28**: 29–33

Hardy JR, Noble T and Smith IE (1989) Symptom relief with moderate dose chemotherapy (mitomycin-C, vinblastine and cisplatin) in advanced non-small cell lung cancer. *Br J Cancer* **60**: 764–766

Harper PG, James LE, Spiro SG and Rudd RM (1997) Five day oral etoposide treatment for advanced small cell lung cancer (SCLC): quality of life (QOL) assessments in a randomised comparison with intravenous chemotherapy [abstract]. *Lung Cancer* (**18** Suppl 1): 204

Hopwood P (1996) Quality of life assessment in chemotherapy trials for non-small cell lung cancer: are theory and practice significantly different? *Semin Oncol* **23**(5 Suppl 10): 60–64

Hopwood P (1997) Evidence for the impact of quality of life. *Lung Cancer* **18**(Suppl 2): 66

Hopwood P, Stephens RJ and Machin D, for the MRC Lung Cancer Working Party (1994) Approaches to the analysis of quality of life data: experiences gained from a Medical Research Council Lung Cancer Working Party palliative chemotherapy trial. *Qual Life Res* **3**: 339–352

Hopwood P and Stephens RJ, on behalf of the Medical Research Council (MRC) Lung Cancer Working Party (1995) Symptoms at presentation for treatment in patients with lung cancer: implications for the evaluation of palliative treatment. *Br J Cancer* **7**: 633–636

Hopwood P, Harvey A, Davies J, Stephens RJ, Girling DJ, Gibson D and Parmar MK (1998) Survey of the administration of quality of life (QL) questionnaires in three multicentre randomised trials in cancer. *Eur J Cancer* **34**(1): 49–57

MRC LCWP (1996*a*) Randomised trial of four-drug vs less intensive two-drug chemotherapy in the palliative treatment of patients with small-cell lung cancer (SCLC) and poor prognosis. *Br J Cancer* **73**: 406–413

MRC LCWP (1996*b*). Randomised trial of palliative two-fraction versus more intensive 13-fraction radiotherapy for patients with inoperable non-small cell lung cancer and good performance status. *Clin Oncol* **8**: 167–75

Noble S and Goa KL (1997) Gemcitabine: a review of its pharmacology and clinical potential in non-small cell lung cancer and pancreatic cancer. *Drugs* **54**: 447–472

Non-small Cell Lung Cancer Collaborative Group (1995) Chemotherapy in non-small cell lung cancer: a meta-analysis using updated data on individual patients from 52 randomised clinical trials. *BMJ* **311**: 899–909

Pocock S and Simon R (1975) Sequential treatment assignment with balancing of prognostic factors in controlled clinical trials. *Biometrics* **31**: 103–115

Stephens RJ, Hopwood P, Girling DJ and Machin D (1997) Randomized trials with quality of life endpoints. Are doctors' ratings of patients' physical symptoms interchangeable with patients' self-ratings? *Qual Life Res* **6**: 225–236

Stephens RJ, Hopwood P and Girling D (1999) Defining and analysing symptom palliation in cancer clinical trials: a deceptively difficult exercise. *Br J Cancer* **79**: 538–544

Stewart LA, Pignon JP, Parmar MKB, Le Chevalier T and Souhami RL, on behalf of the NSCLC Collaborators Group (1994) A meta-analysis using individual patient data from randomised clinical trials (RCTs) of chemotherapy (CT) in non-small cell lung cancer (NSCLC): (3) Survival in the supportive care setting [abstract]. *Proc Am Soc Clin Oncol* **13**: 337

Thatcher N, Anderson H, Betticher DC and Ranson M (1995) Symptomatic benefit from gemcitabine and other chemotherapy in advanced non-small cell lung cancer: changes in performance status and tumour-related symptoms. *Anti-Cancer Drugs* **6**(Suppl 6): 39–48

Thatcher N, Hopwood P and Anderson H (1997) Improving quality of life in patients with non-small cell lung cancer: research experience with gemcitabine. *Eur J Cancer* **33**(Suppl 1): S8–S13

World Health Organization (1979) WHO Handbook for Reporting Results of Cancer Treatment. *World Health Organization* **48**: Geneva

© 2000 Cancer Research Campaign
